# Acute Cholestatic Hepatitis A Virus Infection Presenting with Hemolytic Anemia and Renal Failure: A Case Report

**DOI:** 10.1155/2013/438375

**Published:** 2013-05-08

**Authors:** Robert T. Lapp, Fedja Rochling

**Affiliations:** University of Nebraska Medical Center, 982000 NMC, Omaha, NE 68198, USA

## Abstract

Hepatitis A virus is the most common acute viral hepatitis worldwide with approximately 1.5 million cases annually. Hepatitis A virus infection in general is self-limited. In rare cases, hepatitis A virus infection may cause renal failure, hemolytic anemia, and/or cholestasis. We report the first case of acute cholestatic hepatitis A virus infection complicated by hemolytic anemia, and renal failure in one patient. A 42-year-old Caucasian male presented with cholestasis, hemolytic anemia and renal failure after consuming street tacos in Central and South America while on a business trip. His protracted course required corticosteroid therapy, multiple sessions of plasma exchange, and numerous units of packed red blood cells. This case demonstrates the importance of vaccination in high-risk adults. A prompt diagnosis of acute hepatitis A virus infection is essential, as uncommon presentations may delay diagnosis leading to permanent morbidity and potentially death in fulminant cases. We also demonstrate the efficacy of treatment of cholestatic hepatitis A virus infection, hemolytic anemia, and renal failure with corticosteroids and plasma exchange.

## 1. Introduction

Hepatitis A virus (HAV) is the most common acute viral hepatitis worldwide and in general has a self-limited course [1, 2]. Extrahepatic manifestations of hepatitis A infection are uncommon. Jaundice, dark urine, fatigue, loss of appetite, and nausea are reported in over 80% of patients with HAV [3]. Rarely has renal failure, hemolytic anemia, and cholestasis been described in patients with HAV infection We describe a gentleman with protracted course of cholestatic hepatitis due to HAV infection, complicated by renal failure and hemolytic anemia. 

This case underscores the importance of vaccination in high-risk adults and prompts diagnosis of acute HAV infection, as uncommon presentations may delay diagnosis leading to permanent morbidity and potentially death in fulminant cases. This is the first case of acute cholestatic hepatitis A virus infection with associated hemolytic anemia and renal failure. 

## 2. Case Report

A 42-year-old Caucasian man with no significant past medical history presented with one month of nausea, nonbilious vomiting, intermittent fever, anorexia, and jaundice. His symptoms started one week after eating street tacos while traveling through Mexico, Peru, and Argentina for business. A local physician in Argentina initially treated him with 10 days of amoxicillin and 6 days of methylprednisolone followed by 5 days of azithromycin with minimal improvement. He traveled back to the United States before his fatigue worsened prompting his presentation to our hospital. He consumes 1-2 glasses of wine per week and denied illicit drug use, high-risk sexual encounters, or new tattoos. He did not update his vaccinations prior to his trip.

Clinical examination demonstrated a fever with a temperature of 40°C with pulse of 89/min, respirations of 20/min, and blood pressure of 95/51 mmHg. His physical exam was unremarkable except for scleral icterus and jaundice. Admission laboratory evaluation demonstrated anemia, acute kidney injury, and cholestatic liver injury (Table 1). In addition, his erythrocyte sedimentation rate was 93 mm/hour. 

He was rehydrated, transfused two units of packed red blood cells, and given supportive care. He was administered doxycycline for high fevers. Abdominal ultrasound with the Doppler studies demonstrated a large portal vein, no evidence of biliary obstruction, and spleen measuring 15.5 cm. 

The laboratory evaluation for his cholestatic hepatitis showed that IgM anti-HAV was positive. Further evaluation was negative for hepatitis B virus, hepatitis C virus, primary biliary cirrhosis, Epstein-Barr virus, cytomegalovirus, hemochromatosis, Wilson's disease, alpha-1 antitrypsin deficiency, and antinuclear antibody screen. However, his antismooth muscle antibody (ASMA) screen was elevated at 37 units (normal <19 units). 

Evaluation of his acute kidney injury included urineanalysis demonstrating bilirubin, urobilinogen, trace protein, and hemoglobinuria, but no erythrocytes or eosinophils were present. No kidney abnormalities were present on abdominal ultrasound. 

On day 3 of admission, the total bilirubin and creatinine increased, while the patient remained anemic despite 7 units of packed red blood cells and developed thrombocytopenia (Table 1). Therefore, he underwent hemolysis evaluation demonstrating lactate dehydrogenase of 2194, haptoglobin of <15 (normal >20); thick and thin smears for parasites were negative, fibrinogen 358 mg/dL (240–450), D-dimer 1.4 mcg/mL (0.2–0.4), antithrombin III activity: 65% (80–120%), schistocytes absent on peripheral smear, factor VIII: 326% (50–150%), von Willebrand factor 402% (40–170%) and ADAMTS13 51% (>67%), and negative Coombs' test. Rocky mountain spotted fever, IgG and IgM antibodies, leptospirosis antibody, histoplasmosis antibody and urinary antigen, coccidoidomycosis antibody, parvovirus IgM, and protein electrophoresis were negative. 

**Figure 1:**
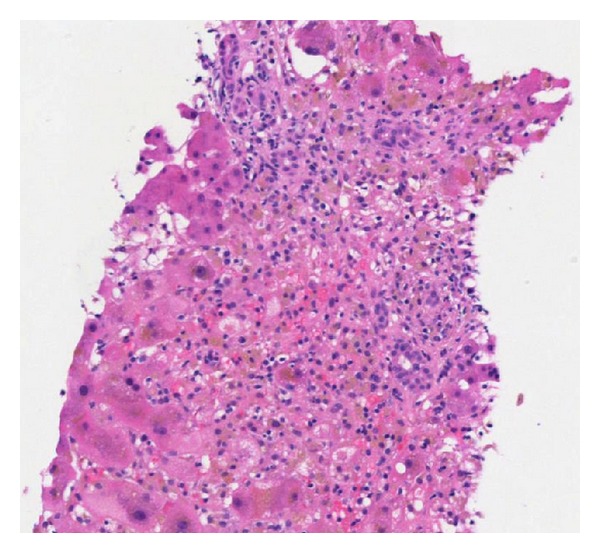
Liver biopsy demonstrating interface hepatitis, cholestasis with brown bile, and hepatocyte dropout.

Plasma exchange was initiated on day 3 of admission given concern for microangiopathic hemolytic anemia versus immune-mediated hemolysis and worsening renal injury in the setting of cholestatic HAV infection. Liver biopsy performed on day 3 of admission demonstrated cholestatic hepatitis with moderate inflammatory activity (grade 3) and areas of pericentral hepatocyte dropout (Figure 1). The liver test elevation was likely from cholestatic HAV infection versus HAV triggered autoimmune process and/or drug induced from amoxicillin and azithromycin. On day 7 of his admission, he was initiated on prednisone therapy as his total bilirubin increased to 31.8 mg/dL and he had ongoing evidence of hemolysis. His total bilirubin peaked on day 9 at 39.8 mg/dL, and continued to decline until his discharge when his total bilirubin was 3.8 mg/dL. 

Despite 6 sessions of plasma exchange and two days of prednisone therapy, he continued to require daily transfusions of packed red blood cells; his reticulocyte indices remained low, and he had evidence of ongoing hemolysis; therefore, he underwent bone marrow biopsy on day 9 of admission. Bone marrow biopsy demonstrated normocellular bone marrow (50–60%) with orderly trilineage hematopoiesis, histiocytosis with phagocytosis, and dyserythropoiesis. Flow cytometry: no phenotypic abnormalities are identified. Peripheral blood smear showed normocytic anemia, thrombocytopenia, and left-shifted granulocytes. It was believed that he likely had a hemolytic anemia with consumptive coagulopathy versus microangiopathic hemolytic anemia versus HAV infection causing his ongoing hemolysis. 

He required a total of 38 units of packed red blood cells during his admission. Upon discharge his hemoglobin was 7.8 g/dL and a platelet count of 161,000. He maintained urine output throughout his hospital stay and never required hemodialysis. He underwent 19 sessions of plasma exchange during his admission. On discharge, his creatinine remained elevated at 1.75. Followup two weeks later in the hepatology clinic demonstrated resolution of his jaundice. He began a prednisone taper at this appointment dropping from 60 mg daily to 30 mg daily and slow taper thereafter. 

## 3. Discussion

Approximately 1.5 million clinical cases of hepatitis A occur worldwide annually but the rate of infection is probably 10 times higher [1]. Hepatitis A infection is generally self-limited; however, it can range from asymptomatic to fulminant hepatitis [4]. Here, we describe a case of cholestatic HAV infection, complicated by renal failure and hemolytic anemia requiring plasma exchange and an extended course of corticosteroids. 

The mean peak of total bilirubin in acute HAV infection is usually less than 10 mg/dL with mean jaundice lasting about 4 weeks [5]. A cholestatic variant of acute HAV has previously described in case reports and small series to occur in 10% of patients with symptomatic disease [6, 7]. These patients typically have aminotransferase levels <500 IU/L, persistent jaundice, intense pruritis, and biochemical evidence of intrahepatic cholestasis, similar to our case. Despite a prolonged course of jaundice, patients typically feel quite well and completely recover on their own. Liver biopsy demonstrates evidence of marked centrilobular cholestasis with mild portal inflammation [8]. This is consistent with our patient's liver biopsy; however, there was pericentral hepatocyte dropout, which was believed secondary to drug-induced liver injury from his previous amoxicillin or azithromycin. Steroid therapy has been postulated to hasten recovery as a therapeutic option in patients with acute cholestatic HAV infection [9, 10]. Our patient was initiated on 60 mg of oral prednisone daily for treatment of his cholestatic hepatitis and hemolytic anemia. He tolerated the prednisone without side effects, and on the day of discharge his total bilirubin decreased to 3.8 mg/dL. 

Hemolytic anemia is an extrahepatic manifestation of viral hepatitis, including HAV. It has been suggested that acute HAV infection induces autoimmune hemolytic anemia [11]. Although the results of the direct Coombs' test were negative; antibodies to ASMA were positive in our patient concerning an autoimmune etiology of his hemolysis. Our patient received plasma exchange and prednisone therapy for treatment of his hemolytic anemia, although the role of plasma exchange in treating autoimmune hemolytic anemia remains uncertain [12]. The patient's last packed red blood cell transfusion was two days prior to discharge. 

Acute renal failure is not uncommon in patients with fulminant hepatitis, and it has been recognized as a rare complication of HAV infection in multiple case reports and small series. The mechanism by which HAV causes renal failure has still not been entirely elucidated, with several mechanisms being hypothesized. Prerenal factors, including loss of appetite, vomiting, and/or diarrhea can cause circulatory insufficiency, activating the rennin-angiotensin system [13, 14]. Hyperbilirubinemia and bile acids may increase the susceptibility of renal tissue to ischemic change [15]. Several studies have demonstrated that bilirubin and bile salts have a direct nephrotoxic effect on the kidneys, resulting in tubular dysfunction and acute tubular necrosis [16]. Another hypothesis involves immune complex-mediated nephritis that may occur during acute hepatitis A. The treatment of renal failure in this setting is usually supportive, and the prognosis is usually favorable; however, permanent renal damage may occur [17]. 

There is a lack of controlled trials evaluating the use of plasma exchange in preventing renal dysfunction in this setting. Given our patient's multiple complications of HAV infection, plasma exchange was initiated. Our patient maintained adequate urine output and did not require hemodialysis during his hospitalization. One month after discharge at his followup in the outpatient hepatology clinic, his creatinine was still elevated at 1.81; however, he maintained high urine output, and he was believed to be dehydrated at this appointment; therefore, there was minimal concern for prolonged renal injury with increased PO intake as an outpatient.

All countries in which our patient traveled were destinations for which HAV prophylaxis is recommended. He is at high risk as he is a frequent traveler to highly endemic countries of HAV. The vaccine against HAV, which provides long-term protection in approximately 95% of patients, provides an opportunity to substantially prevent morbidity and mortality in a cost-effective manner, and our patient should have been vaccinated prior to travel [18]. 

This case demonstrates multiple rare complications of a common worldwide disease including renal failure, cholestasis, and hemolysis. Adequate fluid resuscitation to ensure urine output, avoiding nephrotoxic agents and correcting electrolyte imbalances, is key to treating renal dysfunction in patients with acute HAV. Prompt evaluation of hematological abnormalities is essential as patients with acute HAV are at risk for microangiopathic hemolytic anemia, autoimmune triggered hemolysis, and hemolysis triggered by the hepatitis virus itself. Treatment of hemolysis and cholestasis in these patients is usually supportive; however, certain cases may warrant specific interventions including corticosteroids or plasma exchange, as demonstrated in this case. A universal vaccination program for children, adolescents, and high-risk adults against HAV is the most effective strategy for preventing HAV and its associated complications. This case demonstrates uncommon presentation and complications of a common disease that are typically seen by a wide range of specialties including gastroenterology, hepatology, infectious disease, travel medicine, and primary care physicians.

## Figures and Tables

**Table 1:** Trend in patient's laboratory and transfusion data during his admission.

Labs	Day 1	Day 2	Day 3	Day 7	Day 9	Day 26
WBC (×10*E*3/*µ*L)	3.5	3.5	4.5	7.5	10.9	7.6
Hgb (g/dL)	6.2	7	8.4	6.7	7.9	7.8
Platelet (×10*E*3/*µ*L)	134	112	81	59	110	161
Creatintine (mg/dL)	1.44	1.79	2.21	2.31	2.3	1.75
Total bilirubin (mg/dL)	21.1	17.9	23.4	31.8	39.8	3.8
Direct bilirubin (mg/dL)			14.1	21		2
LDH (U/L)	2194	2247	3223	1988		
AST (IU)	410	395	398	350	281	70
ALT (IU)	315	270	273	222	243	131
Alkaline phosphatase (IU)	75	46	49	37	48	105
INR	1.1	1.3	1.1	1.1	1.1	1
BUN (mg/dL)	26	31	42	86	76	25
Total PRBC transfused to date	2	5	7	18	25	38

## Abbreviations

HAV: Hepatitis A virus
ASMA: Antismooth muscle antibody.
